# Herbst appliance: combination with fully individualized lingual vs conventional buccal braces vs surgery

**DOI:** 10.1186/1746-160X-10-S1-O11

**Published:** 2014-12-12

**Authors:** Ariane Hohoff

**Affiliations:** 1 Department of Orthodontics, University Clinic Münster, Münster, Germany

## 

The Herbst appliance –first presented to the dental community in 1909- is a traditional fixed bite-jumping device aiming at skeletal and/or dentoalveolar correction of class II malocclusions. It is especially indicated in the permanent dentition at or just after the pubertal peak of growth (Pancherz, 1997). An upper age limit for successful treatment with the Herbst device is difficult to define (Pancherz and Ruf, 2008). The oldest patient treated with this appliance was –to the best knowledge of literature by the author– 44.4 years old (Ruf and Pancherz, 2006). The Herbst device can be considered an alternative to orthognathic surgery in borderline adult skeletal class II malocclusions, especially when a great facial improvement is not the main treatment goal (Ruf and Pancherz, 2004).

Orthodontic therapy with fully individualized lingual appliances does not only offer evident aesthetic superiority and a high accuracy (Grauer and Proffit, 2011), but also -and this is decisive in comparison to treatment with conventional braces and wires- *medical advantages* with respect to avoidance of white spot lesions, that progress or develop approximately 5 times less (van der Veen et al., 2010) and in combination with the Herbst appliance:

• with a mean of only 2.2°+/-1° (Wiechmann et al., 2010) as compared to data from literature (El-Fateh und Ruf, 2011) undesired proclination of the lower front teeth is considerably less; due to the exact fit of slot and wire even an uprighting of the mandibular incisors is possible (Wiechmann et al., 2008), if planned in the set up (Wiechmann et al., 2010).

• as compared to data given in literature the mean correction of the axis of the upper incisors and the mean reduction of PAR index are better, the mean reduction of overjet is better or identical (Vu et al., 2012).

• in the event of agenesis of teeth distal to the lower canines and planned orthodontic space closure additional (skeletal) anchorage is unnecessary, as mesialization can be performed against the force vector of the telescopes.

